# Supplementary material for: Linking Molecular Pathways and Large-Scale Computational Modeling to Assess Candidate Disease Mechanisms and Pharmacodynamics in Alzheimer's Disease
Source: Front Comput Neurosci. 2019 Aug 13;13:54. doi: 10.3389/fncom.2019.00054 (PMC6700386; doi:10.3389/fncom.2019.00054)
Supplement: Supplementary file 1 [file Data_Sheet_1.PDF]

SUPPLEMENTARY MATERIAL.
FIGURES.

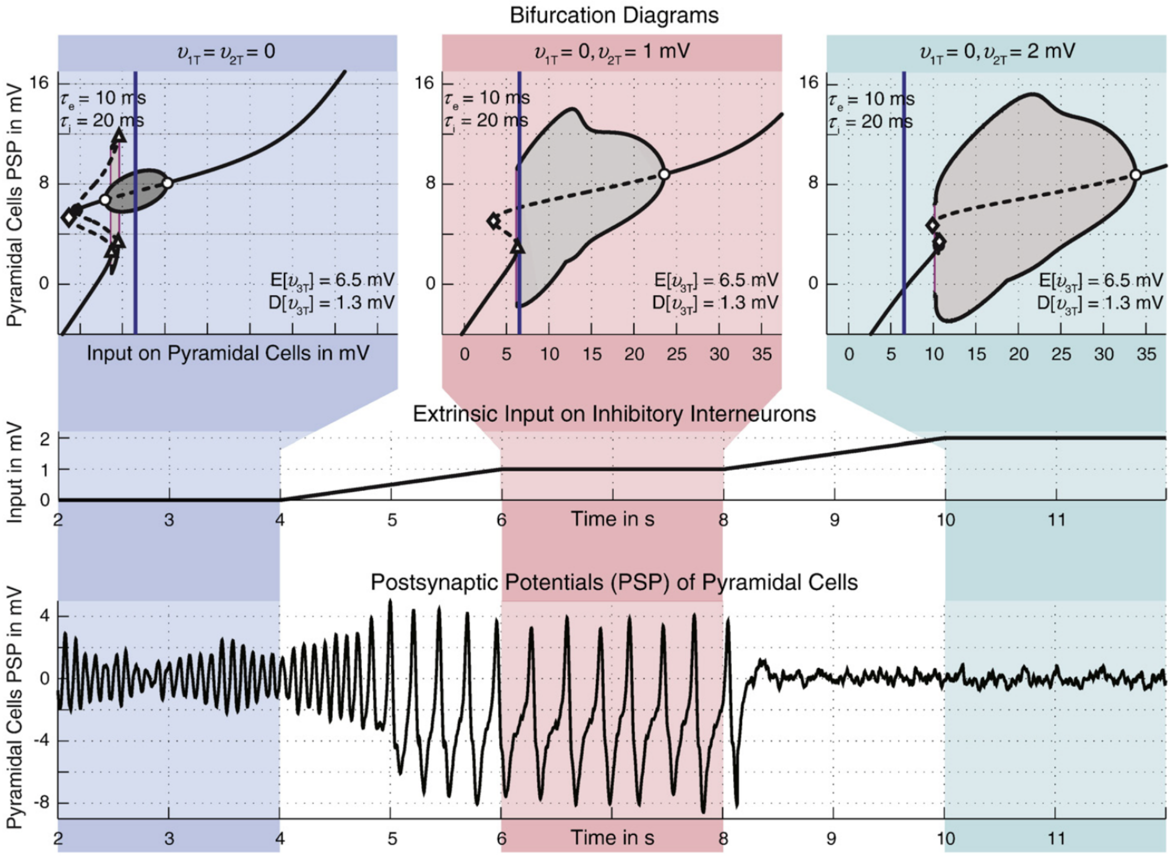

Supplementary Figure 1. Input on pyramidal cells in Jansen-Rit model leads to fundamental changes (criticalities) in the behavior of the system. Taken from Spiegler et al., figure 13 of (1) with permission. Input on inhibitory cells is changed with time (middle row). Shown are three different states of the model: amplitude-modulated alpha activity (highlighted in blue), slower spiking activity (red) and noise in the absence of intrinsic oscillations (green). In the top row, the corresponding bifurcation diagrams are shown for different input values on inhibitory cells. The diagrams show the bifurcations between input on pyramidal cells (x) and the PSP of pyramidal cells (y). Criticalities lead to fundamental changes of the temporal behavior of PSP for only slightly different inputs. In this case, the criticalities of the non-linear system are bifurcations in its mathematical meaning (2). Different types of bifurcations can be seen: sub-critical (black) and supercritical (white) Andronov–Hopf bifurcations, saddle–saddle bifurcations (diamonds) and saddle-node bifurcations (triangles), global bifurcations (red lines). For a more detailed description and for the context of this figure in the corresponding study, please consider the original publication (1).

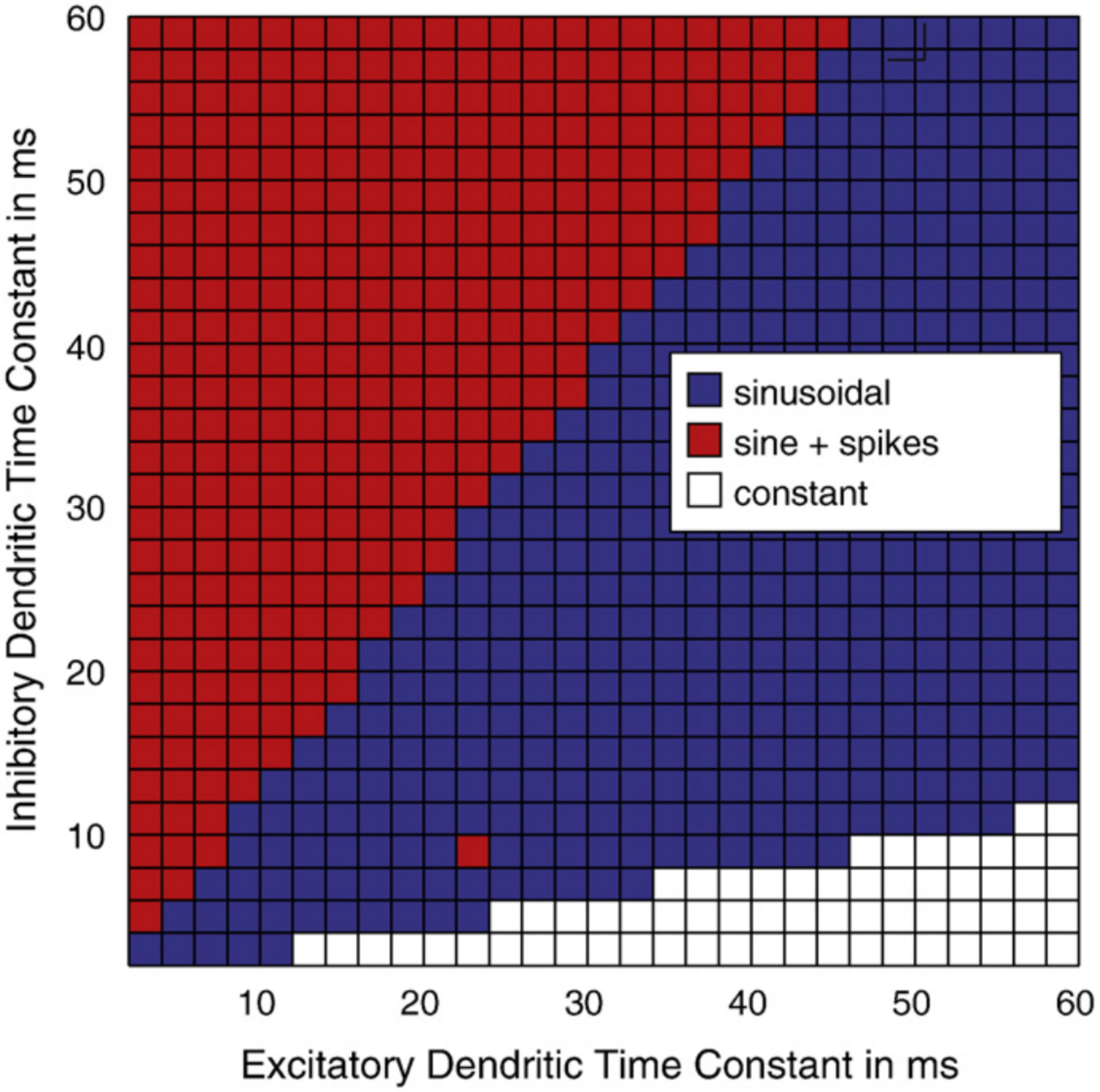

Supplementary Figure 2. Ratio of excitatory and inhibitory time constants modulates frequency in Jansen-Rit model Taken from Spiegler et al., figure 7 of (1) with permission. Blue regions in the parameter space show fast sinusoidal oscillations in alpha rhythm. In red areas, also slower spikes in theta rhythm get possible. For a more detailed description and for the context of this figure in the corresponding study, please consider the original publication (1).

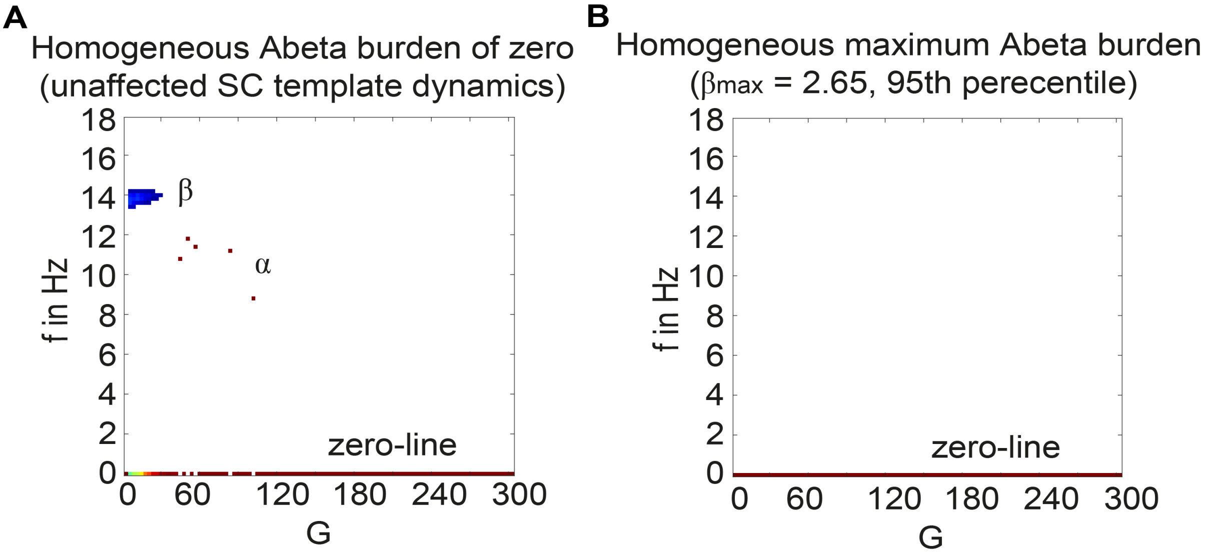

Supplementary Figure 3. Control simulations for the standardized SC template with extreme values of Abeta burden. Because the only individual feature in this study is the Abeta distribution, all participants show the same behaviour for an equal and homogeneous Abeta distribution. Density of regions with a specific dominating frequency at each G. Shown are the sums of all participants regions. **(A)** Homogeneous distribution with Abeta burden of zero at each region, therefore representing the unaffected dynamics that are only driven by the underlying averaged healthy SC template. There is a small beta cluster and single G values between 50 and 100 with alpha rhythms, but the behaviour is dominated by the zero-line. See also **Figure 6A**. **(B)** Homogeneous distribution with Abeta burden of β_{max} = 2.65 at each region. Maximum Abeta was calculated by the 95th percentile of all regions in all participants. It is also represented in the sigmoid curve in **Figure 3**. The full G spectrum is characterized by the zero-line. See also **Figure 6I**.

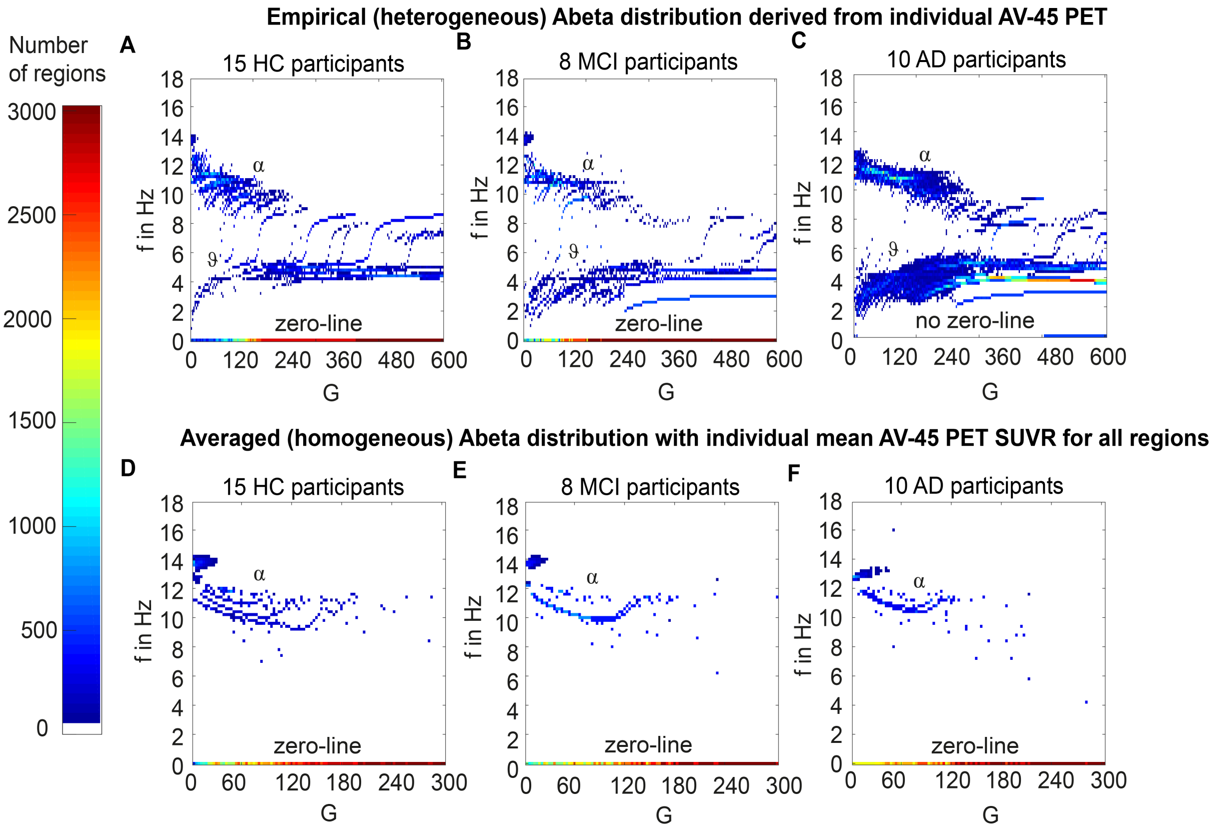

Supplementary Figure 4. AD-specific slowing in neural frequencies and influence of the heterogeneous spatial pattern of Abeta distribution to the spectral behavior. (A, B, C) - Overview of the different frequency behaviors dependent on global coupling factor G, summarized for the three groups HC, MCI, AD**.** The plots show the density of regions with a specific dominating frequency at each G. Shown are the sums of all participants` regions for **(A)** HC (n=15), **(B)** MCI (n=8) and **(C)** AD (n=10). Notably some regions reside at a frequency of 0 beginning at low G values for HC and MCI. This behavior is not apparent for AD, there is only a blue line (meaning low density) at zero at a high G **(C)** – because only 1 out of the 10 AD participants showed a zero-line behavior. **(D, E, F)**: corresponding plots for simulations with homogeneous distribution of averaged Abeta load for each subject, so without spatial information about Abeta distribution and without Abeta heterogeneity. The mean AV-45 PET SUVR for each participant was applied to every region of the brain. The phenomena found in panels **(A-C)** do not appear in **(D)** HC, **(E)** MCI and **(F)** AD. Namely all show a similar synchronized alpha rhythm at low G and convert early to the zero-line behaviour. This strongly supports the hypothesis that not the general burden of Abeta is the driving factor for the observed phenomena in this study. It seems to be more the spatial heterogeneity in the brain, meaning that in the brain coexist areas with different local dynamics that are influencing each other.

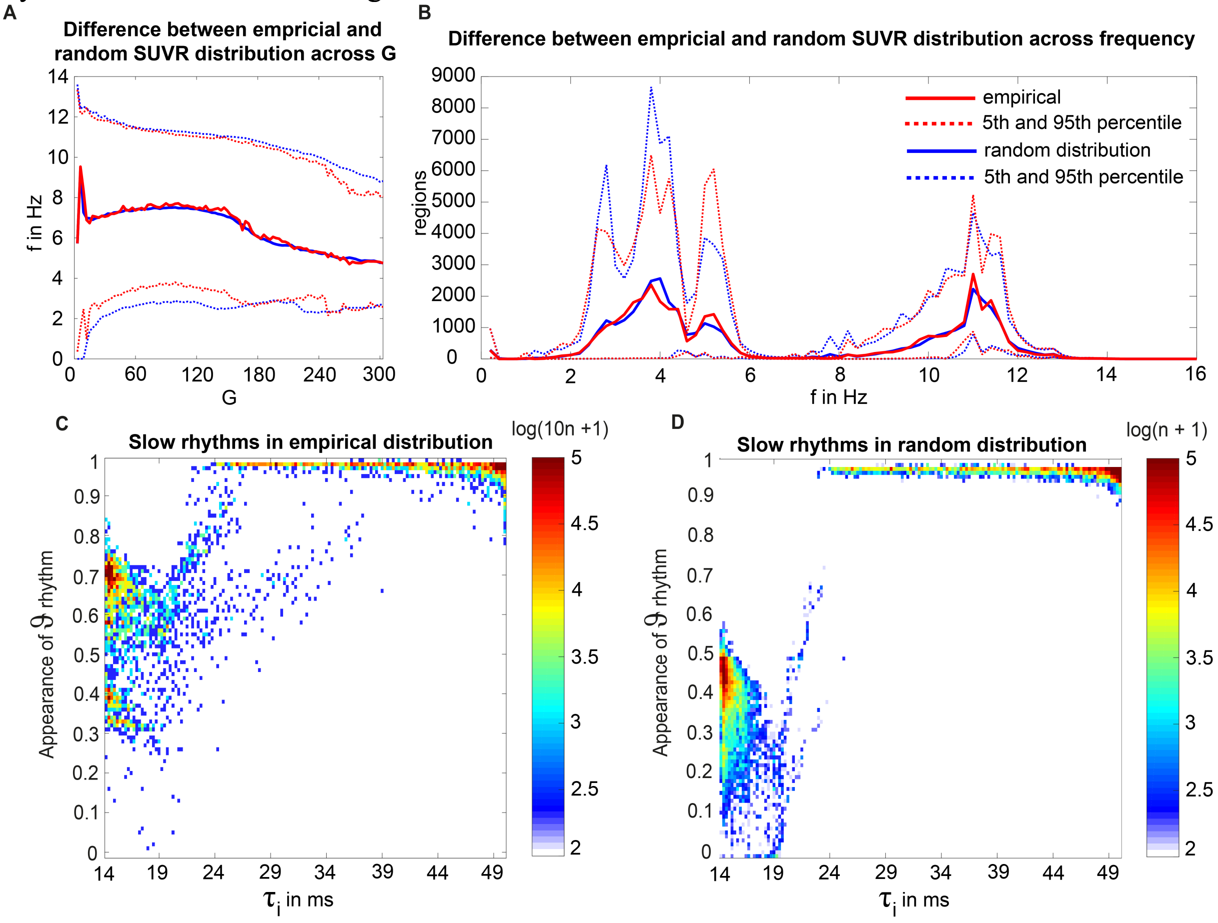

Supplementary Figure 5. Results for random spatial distribution of Abeta PET SUVRs of AD participants. (A) Mean dominating frequencies (y) along global coupling (x), averaged across regions and subjects. Color legend shown in **(B)**. There is no relevant difference except the smother contour of the blue line because of 10-times more simulations. **(B)** Spectrogram-like plot with amount of regions (y) per frequency (x). Again the random distribution and the empirical distribution do not differ. **(C, D)** Dependency between Abeta-PET-derived time constant (x) and the probability of dominant theta rhythm (y) across all simulations. **(C)** empirical distribution, **(D)** random distribution. Again we can observe the

models criticalities at about 18ms and 24ms, where the theta probability increases and alpha rhythms disappear. Moreover, we can see a shift of the sweet spot for low time constants by the ‘shuffling’: meanwhile for τ_i =14ms in the empirical distribution theta appears in 70% of the simulations, in the corresponding simulations with random spatial distribution it appears only in 45% of the simulations. This means that the AD-specific spatial pattern leads to specific slowing for regions with very low Abeta in comparison to the random distribution.

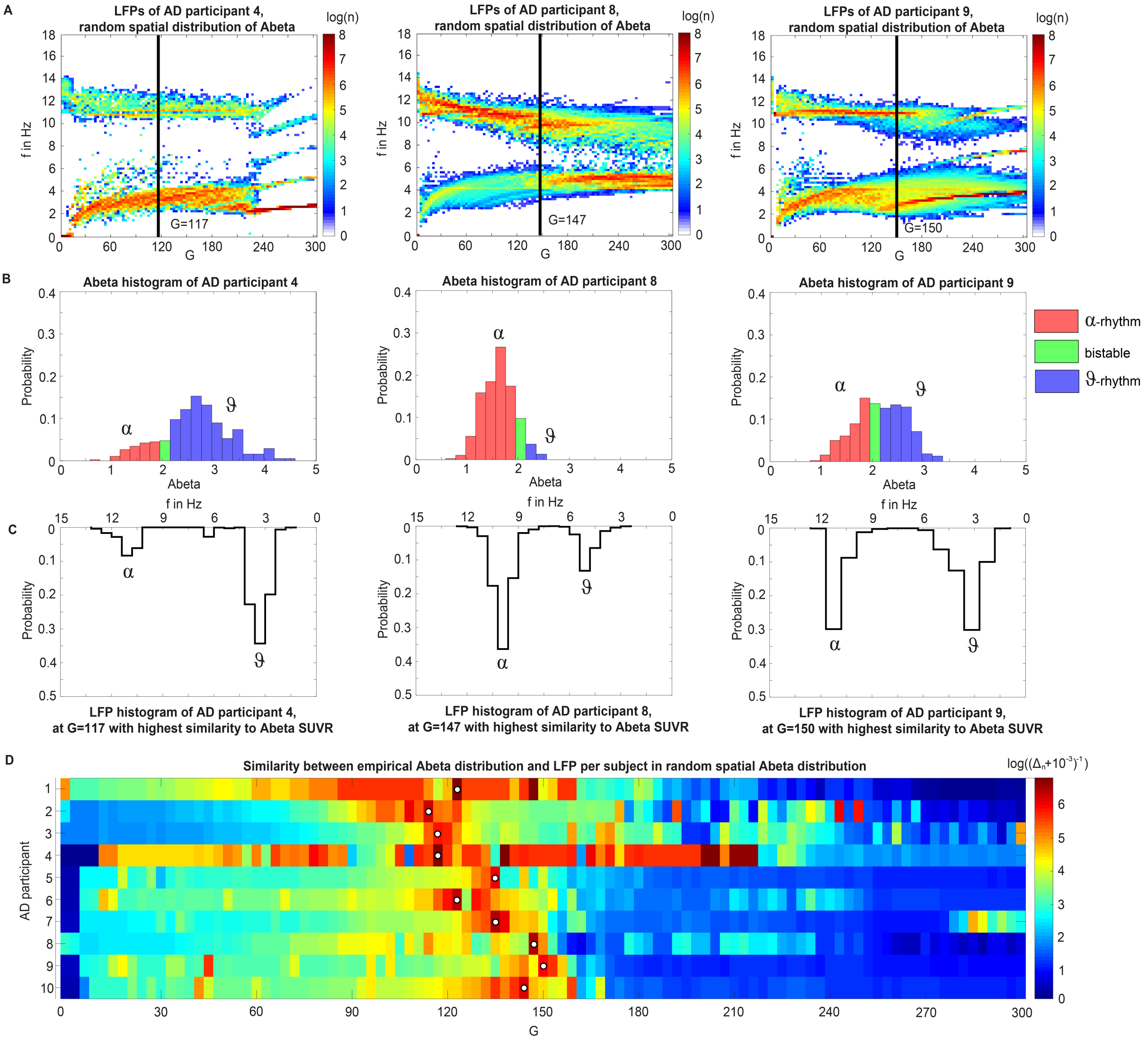

Supplementary Figure 6. Number of regions in different regimes determine overall spectral properties of simulated EEG. Surrogate Abeta PET SUVR distributions reveals the role of region burden for the emergence different dynamical regimes. **(A)** shows for three exemplary AD participants (4, 8 and 9) the dominating frequencies (y) of each region depending on global coupling factor G (x axis). Shown is the sum of 10 random spatial distributions of the empirical Abeta . The corresponding histograms with the normalized amount of regions with various Abeta can be seen in **(B)**: classified by their inhibitory time constant to the alpha, theta or bistable regime (**Figure 6**). Below in **(C)** is the frequency distribution shown for a specific value of G, where the ratio of theta rhythm corresponds best to the ratio of regions in theta regime, indicated by a black line in **(A)**. This similarity is demonstrated in **(D)**: the optimal range (white dot) is very similar for all 10 AD subjects at 100<G<150. Similarity is quantified here as the logarithm of the reciprocal of absolute difference between the ratios of theta to alpha and theta together, meaning that high values show high similarity. Note that subject 4 has three additional optima beside the presented one at G = 117: 138, 201, and 204. Beside this optimal correspondence of frequencies and Abeta

SUVRs, we can see a dependency across large parts of the G spectrum: for participant 4, the high amount of theta regime regions lead to a dominant theta cluster in the full G spectrum and synchronizes to a theta/delta rhythm for higher Gs, independent of the spatial distributions. Vice versa, participant 8 has a high amount of alpha regime regions and shows therefore a dominant alpha cluster until G=150 – afterwards the bistable and theta regions reach the theta limit cycle (see **Figure 6**) and propagate slower rhythms. Participant 9 has an equal distribution of alpha and theta regime regions and many bistable regions, which leads to alpha and theta clusters of the same intensity until G=150, while again afterwards the slower rhythms are dominating. The reason for the dominance of slower rhythms for higher G values can also be found in the bifurcation diagrams of **Figure 6**: while in theta regime **(Figure 6I)** for lower Gs the system is in a stable focus and for higher Gs get to the theta limit cycle, the alpha regime **(Figure 6A)** vice versa starts in alpha limit cycle and ends for higher Gs in a stable focus. Since in the absence of noise the system produces no oscillations in stable focuses, for higher Gs the alpha regions with no intrinsic oscillations can easily be synchronized to the propagated theta signal of neighbored regions. All this indicates that TVB weights the distribution of Abeta and transforms it to a complex slowing phenomenon.

TABLES.

Supplementary Table 1. MPRAGE metadata.

ID	Model	TE [ms]	TR [s]	MatrixSize	VoxelSize [mm]
023_S_1190	TrioTim	2.98	2.3	(176, 240, 256)	(1.0, 1.0, 1.0)
002_S_1280	Prisma_fit	2.95	2.3	(176, 240, 256)	(1.2000046, 1.0546875, 1.0546875)
011_S_4547	Prisma_fit	2.98	2.3	(208, 240, 256)	(1.0, 1.0, 1.0)
168_S_6142	Prisma_fit	2.98	2.3	(208, 240, 256)	(0.9999948, 1.0, 1.0)
002_S_6103	Prisma_fit	2.98	2.3	(208, 240, 256)	(1.0, 1.0, 1.0)
002_S_4654	Prisma_fit	2.95	2.3	(176, 240, 256)	(1.199997, 1.0546875, 1.0546875)
022_S_5004	TrioTim	2.98	2.3	(176, 240, 256)	(1.0, 1.0, 1.0)
003_S_6067	Prisma	2.98	2.3	(208, 240, 256)	(1.0, 1.0, 1.0)
002_S_4229	Prisma_fit	2.98	2.3	(208, 240, 256)	(1.0, 1.0, 1.0)
012_S_6073	Prisma	2.98	2.3	(208, 240, 256)	(1.0, 1.0, 1.0)
002_S_1261	Prisma_fit	2.95	2.3	(176, 240, 256)	(1.2000046, 1.0546875, 1.0546875)
002_S_6009	Prisma_fit	2.95	2.3	(176, 240, 256)	(1.2000046, 1.0546875, 1.0546875)
007_S_4488	Prisma	2.98	2.3	(208, 240, 256)	(1.0, 1.0, 1.0)
003_S_4288	Prisma	2.98	2.3	(208, 240, 256)	(1.0, 1.0, 1.0)
002_S_4213	Prisma_fit	2.98	2.3	(208, 240, 256)	(1.0, 1.0, 1.0)
114_S_6039	Verio	2.98	2.3	(176, 240, 256)	(1.0, 1.0, 1.0)
036_S_4430	Skyra	2.95	2.3	(176, 240, 256)	(1.199997, 1.0546875, 1.0546875)
041_S_4974	Prisma_fit	2.95	2.3	(176, 240, 256)	(1.2000046, 1.0546875, 1.0546875)
007_S_4272	Prisma	2.98	2.3	(208, 240, 256)	(1.0, 1.0, 1.0)
011_S_4827	Prisma_fit	2.98	2.3	(208, 240, 256)	(1.0, 1.0, 1.0)

002_S_6053	Prisma_fit	2.98	2.3	(208, 240, 256)	(1.0, 1.0, 1.0)
003_S_4644	Prisma	2.98	2.3	(208, 240, 256)	(1.0, 1.0, 1.0)
002_S_4799	Prisma_fit	2.95	2.3	(176, 240, 256)	(1.2000046, 1.0546875, 1.0546875)
002_S_0413	Prisma_fit	2.95	2.3	(176, 240, 256)	(1.2000046, 1.0546875, 1.0546875)
114_S_0416	Verio	2.98	2.3	(176, 240, 256)	(1.0, 1.0, 1.0)
002_S_5178	Prisma_fit	2.95	2.3	(176, 240, 256)	(1.199997, 1.0546875, 1.0546875)
002_S_6030	Prisma_fit	2.95	2.3	(176, 240, 256)	(1.2000046, 1.0546875, 1.0546875)
003_S_1122	Prisma	2.98	2.3	(208, 240, 256)	(1.0000056, 1.0, 1.0)
011_S_4893	Prisma_fit	2.98	2.3	(208, 240, 256)	(1.0, 1.0, 1.0)
002_S_1155	Prisma_fit	2.95	2.3	(176, 240, 256)	(1.2000046, 1.0546875, 1.0546875)
036_S_4715	Skyra	2.95	2.3	(176, 240, 256)	(1.2000046, 1.0546875, 1.0546875)
007_S_4387	Prisma	2.98	2.3	(208, 240, 256)	(1.0, 1.0, 1.0)
007_S_4620	Prisma	2.98	2.3	(208, 240, 256)	(1.0, 1.0, 1.0)

Supplementary Table 2. FLAIR metadata.

ID	Model	TE [ms]	TR [s]	MatrixSize	VoxelSize [mm]
023_S_1190	TrioTim	443	4.8	(160, 256, 256)	(1.2000046, 1.0, 1.0)
002_S_1280	Prisma_fit	441	4.8	(160, 256, 256)	(1.2000046, 1.0, 1.0)
011_S_4547	Prisma_fit	441	4.8	(160, 256, 256)	(1.2000046, 1.0, 1.0)
168_S_6142	Prisma_fit	441	4.8	(160, 256, 256)	(1.2000002, 1.0, 1.0)
002_S_6103	Prisma_fit	441	4.8	(160, 256, 256)	(1.199997, 1.0, 1.0)
002_S_4654	Prisma_fit	441	4.8	(160, 256, 256)	(1.199997, 1.0, 1.0)
022_S_5004	TrioTim	439	4.8	(160, 256, 256)	(1.199997, 1.0, 1.0)
003_S_6067	Prisma	441	4.8	(160, 256, 256)	(1.2000046, 1.0, 1.0)
002_S_4229	Prisma_fit	441	4.8	(160, 256, 256)	(1.199997, 1.0, 1.0)
012_S_6073	Prisma	441	4.8	(160, 256, 256)	(1.2000046, 1.0, 1.0)
002_S_1261	Prisma_fit	441	4.8	(160, 256, 256)	(1.2000046, 1.0, 1.0)
002_S_6009	Prisma_fit	441	4.8	(160, 256, 256)	(1.2000046, 1.0, 1.0)
007_S_4488	Prisma	441	4.8	(160, 256, 256)	(1.2000046, 1.0, 1.0)
003_S_4288	Prisma	441	4.8	(160, 256, 256)	(1.199997, 1.0, 1.0)
002_S_4213	Prisma_fit	441	4.8	(160, 256, 256)	(1.199997, 1.0, 1.0)
114_S_6039	Verio	343	4.8	(160, 256, 256)	(1.0, 1.0, 1.0)
036_S_4430	Skyra	441	4.8	(160, 256, 256)	(1.2000046, 1.0, 1.0)
041_S_4974	Prisma_fit	441	4.8	(160, 256, 256)	(1.199997, 1.0, 1.0)
007_S_4272	Prisma	441	4.8	(160, 256, 256)	(1.199997, 1.0, 1.0)
011_S_4827	Prisma_fit	441	4.8	(160, 256, 256)	(1.199997, 1.0, 1.0)

002_S_6053	Prisma_fit	441	4.8	(160, 256, 256)	(1.199997, 1.0, 1.0)
003_S_4644	Prisma	441	4.8	(160, 256, 256)	(1.199997, 1.0, 1.0)
002_S_4799	Prisma_fit	441	4.8	(160, 256, 256)	(1.2000046, 1.0, 1.0)
002_S_0413	Prisma_fit	441	4.8	(160, 256, 256)	(1.2000046, 1.0, 1.0)
114_S_0416	Verio	343	4.8	(160, 256, 256)	(1.0, 1.0, 1.0)
002_S_5178	Prisma_fit	441	4.8	(160, 256, 256)	(1.199997, 1.0, 1.0)
002_S_6030	Prisma_fit	441	4.8	(160, 256, 256)	(1.2000046, 1.0, 1.0)
003_S_1122	Prisma	441	4.8	(160, 256, 256)	(1.2000005, 1.0, 1.0)
011_S_4893	Prisma_fit	441	4.8	(160, 256, 256)	(1.2000046, 1.0, 1.0)
002_S_1155	Prisma_fit	441	4.8	(160, 256, 256)	(1.2000046, 1.0, 1.0)
036_S_4715	Skyra	441	4.8	(160, 256, 256)	(1.2000046, 1.0, 1.0)
007_S_4387	Prisma	441	4.8	(160, 256, 256)	(1.2000046, 1.0, 1.0)
007_S_4620	Prisma	441	4.8	(160, 256, 256)	(1.2000046, 1.0, 1.0)

Supplementary Table 3. DTI metadata (only for HC participants to average the SC template)

ID	Model	Institute	TE [ms]	TR [s]	MatrixSize	VoxelSize [mm, mm, mm, s]	n_Bvecs	Bvals
002_S_1280	Prisma_fit	OHSU_AIRC	56	7.2	(116, 116, 80, 55)	(2.0, 2.0, 2.0, 7.2)	49	[0. 1000.]
002_S_6103	Prisma_fit	OHSU_AIRC	56	7.2	(116, 116, 80, 55)	(2.0, 2.0, 2.0, 7.2)	49	[0. 1000.]
003_S_6067	Prisma	USCINI	56	7.2	(116, 116, 80, 55)	(2.0, 2.0, 2.0, 7.2)	49	[0. 1000.]
002_S_6009	Prisma_fit	OHSU_AIRC	56	7.2	(116, 116, 80, 55)	(2.0, 2.0, 2.0, 7.2)	49	[0. 1000.]
007_S_4488	Prisma	MAYO_CLINIC_MRI_58	71	3.4	(116, 116, 81, 127)	(2.0, 2.0, 2.0, 3.4)	115	[0. 500. 1000. 2000.]
003_S_4288	Prisma	USC_Stevens_Hall_Institute	56	7.2	(116, 116, 80, 55)	(2.0, 2.0, 2.0, 7.2)	49	[0. 1000.]
002_S_4213	Prisma_fit	OHSU_AIRC	56	7.2	(116, 116, 80, 55)	(2.5172415, 2.5172415, 2.0, 7.2)	49	[0. 1000.]
002_S_6053	Prisma_fit	OHSU_AIRC	56	7.2	(116, 116, 80, 55)	(2.0, 2.0, 2.0, 7.2)	49	[0. 1000.]
003_S_4644	Prisma	USCINI	56	7.2	(116, 116, 80, 55)	(2.0, 2.0, 2.0, 7.2)	49	[0. 1000.]
002_S_4799	Prisma_fit	OHSU_AIRC	56	7.2	(116, 116, 80, 55)	(2.0, 2.0, 2.0, 7.2)	49	[0. 1000.]
002_S_0413	Prisma_fit	OHSU_AIRC	56	7.2	(116, 116, 80, 55)	(2.0, 2.0, 2.0, 7.2)	49	[0. 1000.]
002_S_5178	Prisma_fit	OHSU_AIRC	56	7.2	(116, 116, 80, 55)	(2.0, 2.0, 2.0, 7.2)	49	[0. 1000.]
002_S_6030	Prisma_fit	OHSU_AIRC	56	7.2	(116, 116, 80, 55)	(2.0, 2.0, 2.0, 7.2)	49	[0. 1000.]
007_S_4387	Prisma	MAYO_CLINIC_MRI_58	71	3.4	(116, 116, 81, 127)	(2.0, 2.0, 2.0, 3.4)	115	[0. 500. 1000. 2000.]
007_S_4620	Prisma	MAYO_CLINIC_MRI_58	71	3.4	(116, 116, 81, 127)	(2.0, 2.0, 2.0, 3.4)	115	[0. 500. 1000. 2000.]

Supplementary Table 4. AV-45 PET (Abeta) metadata.

ID	Scanner	Model	MatrixSize	VoxelSize [mm]

023_S_1190	Siemens	Biograph6_TruePoint	(160, 160, 96)	(1.5, 1.5, 1.5)
002_S_1280	Philips	GEMINI_TF_TOF_16	(160, 160, 96)	(1.5, 1.5, 1.5)
011_S_4547	Siemens	Biograph40_TruePoint	(160, 160, 96)	(1.5, 1.5, 1.5)
168_S_6142	GE	Discovery_STE	(160, 160, 96)	(1.5, 1.5, 1.5)
002_S_6103	Philips	GEMINI_TF_TOF_16	(160, 160, 96)	(1.5, 1.5, 1.5)
002_S_4654	Philips	GEMINI_TF_TOF_16	(160, 160, 96)	(1.5, 1.5, 1.5)
022_S_5004	Philips	Ingenuity_TF_PET_CT	(160, 160, 96)	(1.5, 1.5, 1.5)
003_S_6067	Siemens	Biograph64_TruePoint	(160, 160, 96)	(1.5, 1.5, 1.5)
002_S_4229	Philips	GEMINI_TF_TOF_16	(160, 160, 96)	(1.5, 1.5, 1.5)
012_S_6073	GE	Discovery_710	(160, 160, 96)	(1.5, 1.5, 1.5)
002_S_1261	Philips	GEMINI_TF_TOF_16	(160, 160, 96)	(1.5, 1.5, 1.5)
002_S_6009	Philips	GEMINI_TF_TOF_16	(160, 160, 96)	(1.5, 1.5, 1.5)
007_S_4488	GE	Discovery_690	(160, 160, 96)	(1.5, 1.5, 1.5)
003_S_4288	Siemens	Biograph64_TruePoint	(160, 160, 96)	(1.5, 1.5, 1.5)
002_S_4213	Philips	GEMINI_TF_TOF_16	(160, 160, 96)	(1.5, 1.5, 1.5)
114_S_6039	Philips	GEMINI_TF_TOF_64	(160, 160, 96)	(1.5, 1.5, 1.5)
036_S_4430	Siemens	Biograph40_TruePoint	(160, 160, 96)	(1.5, 1.5, 1.5)
041_S_4974	Siemens	HR+	(160, 160, 96)	(1.5, 1.5, 1.5)
007_S_4272	GE	Discovery_690	(160, 160, 96)	(1.5, 1.5, 1.5)
011_S_4827	Siemens	Biograph40_TruePoint	(160, 160, 96)	(1.5, 1.5, 1.5)
002_S_6053	Philips	GEMINI_TF_TOF_16	(160, 160, 96)	(1.5, 1.5, 1.5)
003_S_4644	Siemens	Biograph64_TruePoint	(160, 160, 96)	(1.5, 1.5, 1.5)
002_S_4799	Philips	GEMINI_TF_TOF_16	(160, 160, 96)	(1.5, 1.5, 1.5)
002_S_0413	Philips	GEMINI_TF_TOF_16	(160, 160, 96)	(1.5, 1.5, 1.5)
114_S_0416	Philips	GEMINI_TF_TOF_64	(160, 160, 96)	(1.5, 1.5, 1.5)
002_S_5178	Philips	GEMINI_TF_TOF_16	(160, 160, 96)	(1.5, 1.5, 1.5)
002_S_6030	Philips	GEMINI_TF_TOF_16	(160, 160, 96)	(1.5, 1.5, 1.5)
003_S_1122	Siemens	Biograph64_TruePoint	(160, 160, 96)	(1.5, 1.5, 1.5)
011_S_4893	Siemens	Biograph40_TruePoint	(160, 160, 96)	(1.5, 1.5, 1.5)
002_S_1155	Philips	GEMINI_TF_TOF_16	(160, 160, 96)	(1.5, 1.5, 1.5)
036_S_4715	Siemens	Biograph40_TruePoint	(160, 160, 96)	(1.5, 1.5, 1.5)
007_S_4387	GE	Discovery_690	(160, 160, 96)	(1.5, 1.5, 1.5)
007_S_4620	GE	Discovery_690	(160, 160, 96)	(1.5, 1.5, 1.5)

Supplementary Table 5. AV-14-51 PET (Tau) metadata

ID	Scanner	Model	MatrixSize	VoxelSize [mm]
023_S_1190	Siemens	Biograph6_TruePoint	(160, 160, 96)	(1.5, 1.5, 1.5)
002_S_1280	Philips	GEMINI_TF_TOF_16	(160, 160, 96)	(1.5, 1.5, 1.5)
011_S_4547	Siemens	Biograph40_TruePoint	(160, 160, 96)	(1.5, 1.5, 1.5)
168_S_6142	GE	Discovery_STE	(160, 160, 96)	(1.5, 1.5, 1.5)
002_S_6103	Philips	GEMINI_TF_TOF_16	(160, 160, 96)	(1.5, 1.5, 1.5)
002_S_4654	Philips	GEMINI_TF_TOF_16	(160, 160, 96)	(1.5, 1.5, 1.5)
022_S_5004	Philips	Ingenuity_TF_PET_CT	(160, 160, 96)	(1.5, 1.5, 1.5)
003_S_6067	Siemens	Biograph64_TruePoint	(160, 160, 96)	(1.5, 1.5, 1.5)
002_S_4229	Philips	GEMINI_TF_TOF_16	(160, 160, 96)	(1.5, 1.5, 1.5)
012_S_6073	GE	Discovery_710	(160, 160, 96)	(1.5, 1.5, 1.5)
002_S_1261	Philips	GEMINI_TF_TOF_16	(160, 160, 96)	(1.5, 1.5, 1.5)
002_S_6009	Philips	GEMINI_TF_TOF_16	(160, 160, 96)	(1.5, 1.5, 1.5)
007_S_4488	GE	Discovery_690	(160, 160, 96)	(1.5, 1.5, 1.5)
003_S_4288	Siemens	Biograph64_TruePoint	(160, 160, 96)	(1.5, 1.5, 1.5)
002_S_4213	Philips	GEMINI_TF_TOF_16	(160, 160, 96)	(1.5, 1.5, 1.5)
114_S_6039	Philips	GEMINI_TF_TOF_64	(160, 160, 96)	(1.5, 1.5, 1.5)
036_S_4430	Siemens	Biograph40_TruePoint	(160, 160, 96)	(1.5, 1.5, 1.5)
041_S_4974	Siemens	HR+	(160, 160, 96)	(1.5, 1.5, 1.5)
007_S_4272	GE	Discovery_690	(160, 160, 96)	(1.5, 1.5, 1.5)
011_S_4827	Siemens	Biograph40_TruePoint	(160, 160, 96)	(1.5, 1.5, 1.5)
002_S_6053	Philips	GEMINI_TF_TOF_16	(160, 160, 96)	(1.5, 1.5, 1.5)
003_S_4644	Siemens	Biograph64_TruePoint	(160, 160, 96)	(1.5, 1.5, 1.5)
002_S_4799	Philips	GEMINI_TF_TOF_16	(160, 160, 96)	(1.5, 1.5, 1.5)
002_S_0413	Philips	GEMINI_TF_TOF_16	(160, 160, 96)	(1.5, 1.5, 1.5)
114_S_0416	Philips	GEMINI_TF_TOF_64	(160, 160, 96)	(1.5, 1.5, 1.5)
002_S_5178	Philips	GEMINI_TF_TOF_16	(160, 160, 96)	(1.5, 1.5, 1.5)
002_S_6030	Philips	GEMINI_TF_TOF_16	(160, 160, 96)	(1.5, 1.5, 1.5)
003_S_1122	Siemens	Biograph64_TruePoint	(160, 160, 96)	(1.5, 1.5, 1.5)
011_S_4893	Siemens	Biograph40_TruePoint	(160, 160, 96)	(1.5, 1.5, 1.5)
002_S_1155	Philips	GEMINI_TF_TOF_16	(160, 160, 96)	(1.5, 1.5, 1.5)
036_S_4715	Siemens	Biograph40_TruePoint	(160, 160, 96)	(1.5, 1.5, 1.5)
007_S_4387	GE	Discovery_690	(160, 160, 96)	(1.5, 1.5, 1.5)
007_S_4620	GE	Discovery_690	(160, 160, 96)	(1.5, 1.5, 1.5)

Supplementary Table 6. Dates of Imaging and MMSE.

ID	MMSE date	MPRAGE date	FLAIR date	DTI date	AV-45 PET date	AV-1451 PET date
023_S_1190	17/11/13	17/10/23	17/10/23		17/10/25	17/11/08
002_S_1280	18/3/7	17/3/13	17/3/13	17/3/13	17/3/02	18/3/5
011_S_4547	17/8/18	17/8/18	17/8/18		17/8/30	17/8/24
168_S_6142	17/12/5	17/12/18	17/12/18		18/1/17	18/1/3
002_S_6103	17/10/25	17/11/20	17/11/20	17/11/20	17/11/21	18/1/17
002_S_4654	18/5/15	17/5/3	17/5/3		17/5/2	18/5/22
022_S_5004	18/6/29	18/3/14	17/3/21		17/3/21	17/4/5
003_S_6067	17/12/4	17/8/18	17/8/18	17/8/18	17/10/13	17/10/18
002_S_4229	18/5/14	17/9/20	17/9/20		17/9/20	17/10/3
012_S_6073	17/9/18	17/9/22	17/9/22		17/10/12	17/10/11
002_S_1261	18/3/8	17/3/15	17/3/15		17/3/14	17/3/15
002_S_6009	17/4/1	17/4/17	17/4/17	17/4/17	17/5/16	17/5/15
007_S_4488	18/6/11	17/9/12	17/9/12	17/9/12	17/9/22	17/9/13
003_S_4288	17/10/2	17/10/3	17/10/3	17/10/3	17/10/3	18/2/22
002_S_4213	17/8/16	17/8/14	17/8/14	17/8/14	17/8/14	17/8/17
114_S_6039	17/8/10	17/7/21	17/7/21		17/8/24	17/10/4
036_S_4430	17/11/15	17/11/07	17/11/07		17/11/15	17/11/21
041_S_4974	17/10/30	17/10/5	17/10/5		17/8/24	17/10/12
007_S_4272	18/1/18	18/1/16	18/1/16		17/12/19	18/1/17
011_S_4827	17/8/24	17/8/31	17/8/31		17/8/28	17/9/7
002_S_6053	17/7/21	17/7/18	17/7/18	17/7/18	17/8/23	17/8/24
003_S_4644	17/6/26	17/6/21	17/6/21	17/6/21	18/2/28	18/4/17
002_S_4799	18/6/7	17/5/22	17/5/22	17/5/22	17/5/18	18/6/13
002_S_0413	17/6/16	17/6/21	17/6/21	17/6/21	17/6/15	17/6/21
114_S_0416	18/7/24	17/10/24	17/10/24		17/10/24	17/11/21
002_S_5178	17/6/6	17/5/31	17/5/31	17/5/31	17/6/5	17/5/31
002_S_6030	17/6/9	17/6/15	17/6/15	17/6/15	17/7/25	17/7/24
003_S_1122	18/7/25	17/5/18	17/5/18		17/8/8	17/8/10
011_S_4893	18/7/17	17/11/8	17/11/8		17/11/1	17/11/7
002_S_1155	18/5/9	17/4/24	17/4/24		17/4/20	17/4/24
036_S_4715	17/10/13	17/10/10	17/10/10		17/10/10	17/10/12
007_S_4387	17/10/31	17/11/1	17/11/1	17/11/1	17/10/24	17/11/29
007_S_4620	17/12/12	17/12/05	17/12/05	17/12/05	17/12/06	17/12/14

Supplementary Table 7. Participants IDs used in this study and corresponding ADNI official ID.

	ID	ADNI ID
**AD**	1	023_S_1190
	2	011_S_4547
	3	168_S_6142
	4	114_S_6039
	5	036_S_4430
	6	041_S_4974
	7	011_S_4827
	8	114_S_0416
	9	011_S_4893
	10	036_S_4715
**HC**	11	002_S_1280
	12	002_S_6103
	13	003_S_6067
	14	002_S_6009
	15	007_S_4488
	16	003_S_4288
	17	002_S_4213
	18	002_S_6053
	19	003_S_4644
	20	002_S_4799
	21	002_S_0413
	22	002_S_5178
	23	002_S_6030
	24	007_S_4387
	25	007_S_4620
**MCI**	26	002_S_4654
	27	022_S_5004
	28	002_S_4229
	29	012_S_6073
	30	002_S_1261
	31	007_S_4272
	32	003_S_1122
	33	002_S_1155

Supplementary Table 8. Overview of the used statistical tests in this study.

**Statistical question (Figure)**	**Data**	**Independent (grouping) variable**	**Statistical Test**	**Justification**
Do specific frequency bands in LFP differ between diagnostic groups for the empirical Abeta distribution? (**Figure 7A**)	Amount of regions with a specific frequency range, averaged over participants (group-wise) and all G values	1. HC and MCI (n=23) 2. AD and HC (n=25) 3. AD and MCI (n=18)	Kruskal-Wallis-Test	Variable does not meet the normality assumption of one-sided ANOVA
Do specific frequency bands in LFP differ between diagnostic groups for the homogeneous Abeta distribution? (**Figure 7B**)	Amount of regions with a specific frequency range, averaged over participants (group-wise) and all G values	1. HC and MCI (n=23) 2. AD and HC (n=25) 3. AD and MCI (n=18)	Kruskal-Wallis-Test	Variable does not meet the normality assumption of one-sided ANOVA
Does the mean frequency in EEG differ between diagnostic groups for the empirical Abeta distribution? (**Figure 7C**)	Mean EEG frequency, averaged over all channels and all participants (group-wise) for different values of G	1. HC and MCI (n=23) 2. AD and HC (n=25) 3. AD and MCI (n=18)	Kruskal-Wallis-Test	Variable does not meet the normality assumption of one-sided ANOVA
Does the mean frequency in LFP differ between diagnostic groups for the	Mean LFP frequency, averaged over all regions and all participants (group-wise)	1. HC and MCI (n=23) 2. AD and	Kruskal-Wallis-Test	Variable does not meet the normality assumption of one-

empirical Abeta distribution? (**Figure 7D**)	for different values of G	HC (n=25)<br>3. AD and MCI (n=18)		sided ANOVA
In how far is the regional LFP frequency linear dependent on the regional Abeta burden? (**Figure 8**)	Regional LFP frequency and regional beta PET SUVR for all regions and all G values, averaged over all participants (group-wise)	1. HC (n=15)<br>2. MCI (n=8)<br>3. AD (n=10)	Linear regression and correlation analysis	Question on linear dependency (given by p) and strength of its dependency (given by R^2)
In how far is there a linear dependency between the logarithmized structural degree and the local Abeta burden? (**Figure 10B**)	Natural logarithm of the structural degree of the averaged healthy standard connectome per region and local Abeta PET SUVR per region averaged over AD participants	AD (n=10)	Linear regression and correlation analysis	Question on linear dependency and strength of its dependency
In how far is there a linear dependency between the logarithmized structural degree and the local probability of theta rhythm in simulations with empirical Abeta distribution? (**Figure 10D**)	Natural logarithm of the structural degree of the averaged healthy standard connectome per region and the proportion of each regions simulations (201 for different values of G for 10 subjects) with dominant theta rhythm (simplified as a frequency that is below 8 Hz and not zero), averaged over AD participants	AD (n=10)	Linear regression and correlation analysis	Question on linear dependency and strength of its dependency
In how far is there a linear dependency between the logarithmized structural degree and the local probability of theta rhythm in simulations with random Abeta distribution? (**Figure 10F**)	Natural logarithm of the structural degree of the averaged healthy standard connectome per region and the proportion of each regions simulations (random Abeta distribution) with dominant theta rhythm, averaged over AD participants	AD (n=10)	Linear regression and correlation analysis	Question on linear dependency and strength of its dependency

1. Spiegler A, Kiebel SJ, Atay FM, Knösche TR. Bifurcation analysis of neural mass models: Impact of extrinsic inputs and dendritic time constants. NeuroImage. 2010;52(3):1041-58.
2. Strogatz SH. Nonlinear dynamics and chaos : with applications to physics, biology, chemistry, and engineering: Second edition. Boulder, CO : Westview Press, a member of the Perseus Books Group, [2015]; 2015.
